# Studying the Adhesion Force and Glass Transition of Thin Polystyrene Films by Atomic Force Microscopy

**DOI:** 10.1186/s11671-017-2426-9

**Published:** 2018-01-09

**Authors:** Hua Kang, Xiaoqin Qian, Li Guan, Meining Zhang, Qiang Li, Aoli Wu, Mingdong Dong

**Affiliations:** 1 Department of Chemistry, Renmin University of China, Beijing, 100872 People’s Republic of China; 2 Interdisciplinary Nanoscience Center (iNANO), Aarhus University, Dk-8000 Aarhus C, Denmark; 3 Jiangsu CASRS Pollution Control Engineering Co., Ltd., Yancheng, People’s Republic of China

**Keywords:** Adhesion Force, Glass Transition Temperature, Polystyrene, Atomic Force Microscope (AFM); Force Distance Curves

## Abstract

The relaxation behaviors of thin polymer films show a strong dependence on temperature and film thickness. Direct quantitative detection of the relaxation behaviors of thin polymer films at nanometer scale by traditional instruments is however challenging. In this study, we employed atomic force microscopy (AFM)-based force-distance curve to study the relaxation dynamics and the film thickness dependence of glass transition temperature (*T*_*g*_) for normal thin polystyrene (PS) films supported on silicon substrate. The adhesion force (*F*_ad_) between AFM tip and normal thin PS film surfaces was quantitatively detected in situ under the variation of temperature and film thickness. The *T*_*g*_ of normal thin PS film was successfully obtained by the abrupt variation of *F*_ad_ under temperature stimulation. Our result showed that the *T*_*g*_ of normal thin PS films decreased with the decreasing film thickness. The study here could be beneficial for understanding the relaxation dynamics of normal thin polymer films.

## Background

The emergence of nanoscience and nanotechnology leads to a large number of usage of polymer films with thickness at the nanometer scale [1]. Previous studies have shown that the properties of thin polymer films differ greatly from that of bulk material due to the size confinement effect [2–4]. The glass transition temperature (*T*_*g*_) of polymer films decreases with decreasing film thickness [5, 6], which could cause the thin polymer films begin to relax at a temperature far below the value for bulk metric [1]. Relaxation behavior and *T*_*g*_ depression with film thickness decreasing in thin polymer films have limited their applications in many cases. For example, when thin polymer films are employed as dielectrics in micro or nano-devices, the dielectric loss could occur far before the breakdown of the thin polymer films [7]. Therefore, quantitative study of the relaxation properties for thin polymer films at nanometer scale is of great importance for their application in nanoscience and nanotechnology.

Atomic force microscopy (AFM) is widely used for measuring surface morphology, mechanical, electrical, and magnetic properties of nanostructured materials [8, 9] and monitoring chemical changes over surfaces [10, 11] due to the benefits of nanometer spatial resolution and high sensitivity. Zhao et al. studied the charge-induced local dewetting of the polymer electrets with charge patterns by monitoring the surface morphology variations using AFM [12]. The *T*_*g*_ depression was also observed by utilizing the patterned charges as an indicator using electric force microscopy (EFM) [13]. Yang et al. using AFM measured the viscosity of unentangled, short-chain polystyrene (PS) films on silicon substrate at different temperatures and found that the transition temperature for the viscosity decreased with the decreasing film thickness [14].

Relaxation dynamics and *T*_*g*_ depression of thin films with the decreasing film thickness are closely related to the mechanical properties of polymer films, such as friction, adhesion, elastic, and viscoelastic properties [15]. These mechanical properties of polymer films show a strong dependence on the temperature and the film thickness. Hammerschmidt et al. probed the viscoelastic relaxation of thin polymer films with temperature-controlled friction force microscopy (FFM), and the results showed that the peak in the viscoelasticity dependence of friction was attributed to the glass-to-rubber transition [2, 16]. Akabori et al. studied the surface relaxation behaviors in PS films with different thicknesses by lateral force microscopy (LFM) [17]. Related references also reported that the *T*_*g*_ of polymers could be determined by AFM, particularly through the acquisition of force-distance curves. For example, Cappella et al. studied the *T*_*g*_ of amorphous polymer and their elastic-plastic properties as a function of temperature using AFM based force-distance curves [18], and the whole Young modulus as well as the yield strength in the vicinity of *T*_*g*_ was characterized. Bliznyuk et al. measured the surface *T*_*g*_ of PS with different molecular weights by force-distance measurements using scanning force microscopy (SFM). The results showed that the surface *T*_*g*_ depression was mainly caused by polymer chain entanglement variation [19]. The quantities including stiffness, hysteresis, and pull-off force which were calculated from the force-displacement curves captured at different temperatures obviously change at the vicinity of *T*_*g*_ [19]. In addition, Wang et al. investigated the surface dynamics of ultrathin poly (tert-butyl acrylate) (PtBuA) films and observed the variation of surface chain mobility with film thickness changing by atomic force microscopic adhesion measurement (AFMAM) [20].

In view of the fact that the AFM tip is very sensitive to weak forces, it could probe the adhesion force interaction, which is difficult to be detected by other instruments [21]. Therefore, AFM in this way is a significantly direct and more sensitive technique to study surface relaxation properties. In this work, we studied the relaxation dynamics and the film thickness dependence of *T*_*g*_ for normal thin PS films by AFM force-distance mode. The adhesion force (*F*_ad_) between the AFM tip and thin PS film surfaces was quantitatively detected in situ under the stimulation of temperature and the variation of film thickness.

## Methods

### Materials

All materials and chemicals were purchased commercially and used as received. PS (Mw = 4000) was purchased from Alfa Aesar, and chlorobenzene was purchased from Sinopharm Chemical Reagent Beijing Co. Single-side polished silicon wafer was purchased from Silicon Quest International. Thin PS films with various thicknesses from 18 to 127 nm were prepared on silicon wafer using spin-coating from chlorobenzene solutions of PS. Film thickness was controlled by changing the concentration of the PS solution and spin-coating rates. The spin-casted films were annealed at 358 K for 2 h, and film thicknesses were measured using AFM.

### Instruments

The force-distance curves and adhesion forces were recorded using a Dimension Icon system (Bruker, USA). A V-shaped silicon nitride AFM tip with a nominal spring constant (*k* ≈ 0.1 N·m^−1^) was used. Contact mode AFM was employed to monitor the adhesion forces in situ.

### Adhesion Force Measurements

**Fig. 1:**
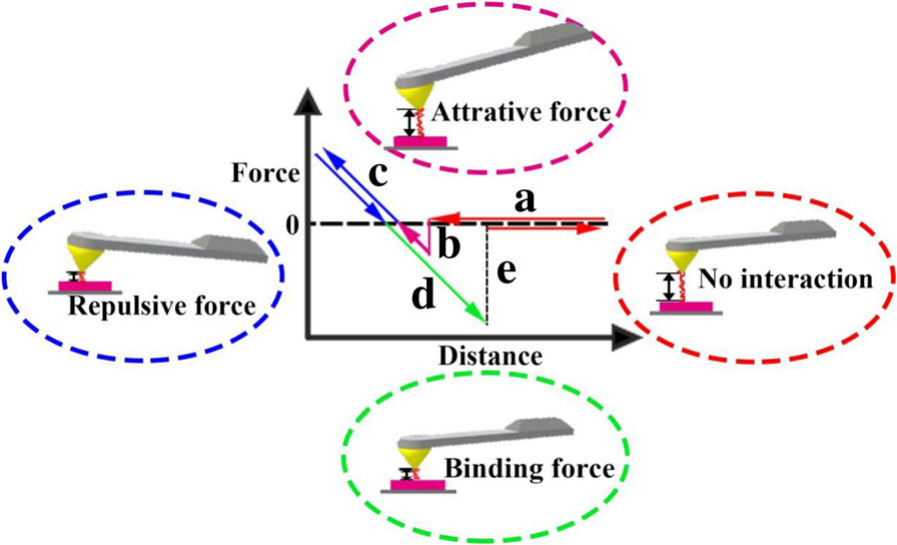
Schematic illustration of adhesion force measurement for normal thin polymer films supported on silicon substrate. The AFM tip **a** firstly approaches the sample surface at a discrete distance above the sample, **b** continues to approach until the tip touches the sample surface, **c** begins to deform the sample surface under a load force and shows a small indentation and **d**–**e** withdraws from the sample surface

The schematic diagram shown in Fig. 1 illustrates the process of adhesion force measurement. The horizontal and vertical axes are the vertical distance between the tip and sample (*z*) and the applied load (*F*), respectively. The pull-off force is assumed to be *F*_ad_, which results in the separation between the tip and the sample. For each tip-sample interaction circle, the AFM tip firstly approaches the sample surface at a discrete distance above the sample, and there is no interaction between the tip and the sample surface (Fig. 1a). The AFM tip continues to approach until the tip touches the sample surface with an attractive force between the tip and the sample surface, as shown in Fig. 1b. Then, the AFM tip begins to deform the sample surface under the load force and shows a small indentation, which is derived from the part of the repulsive force region of the force curves (Fig. 1c). When the tip withdraws from the sample surface, the binding force between the tip and the sample surface makes the AFM tip deform the sample surface in the opposite direction and finally detaches from the surface (Fig. 1d, e).

The *F*_ad_ measurements were conducted under the cooling down process from a temperature higher than the *T*_*g*_ of bulk materials with a cooling down rate of 2 K/min. The relative humidity is controlled below 10% since the capillary menisci formed between the tip and the film surface could contribute to the measured forces [22].

### Modulus Measurements

**Fig. 2:**
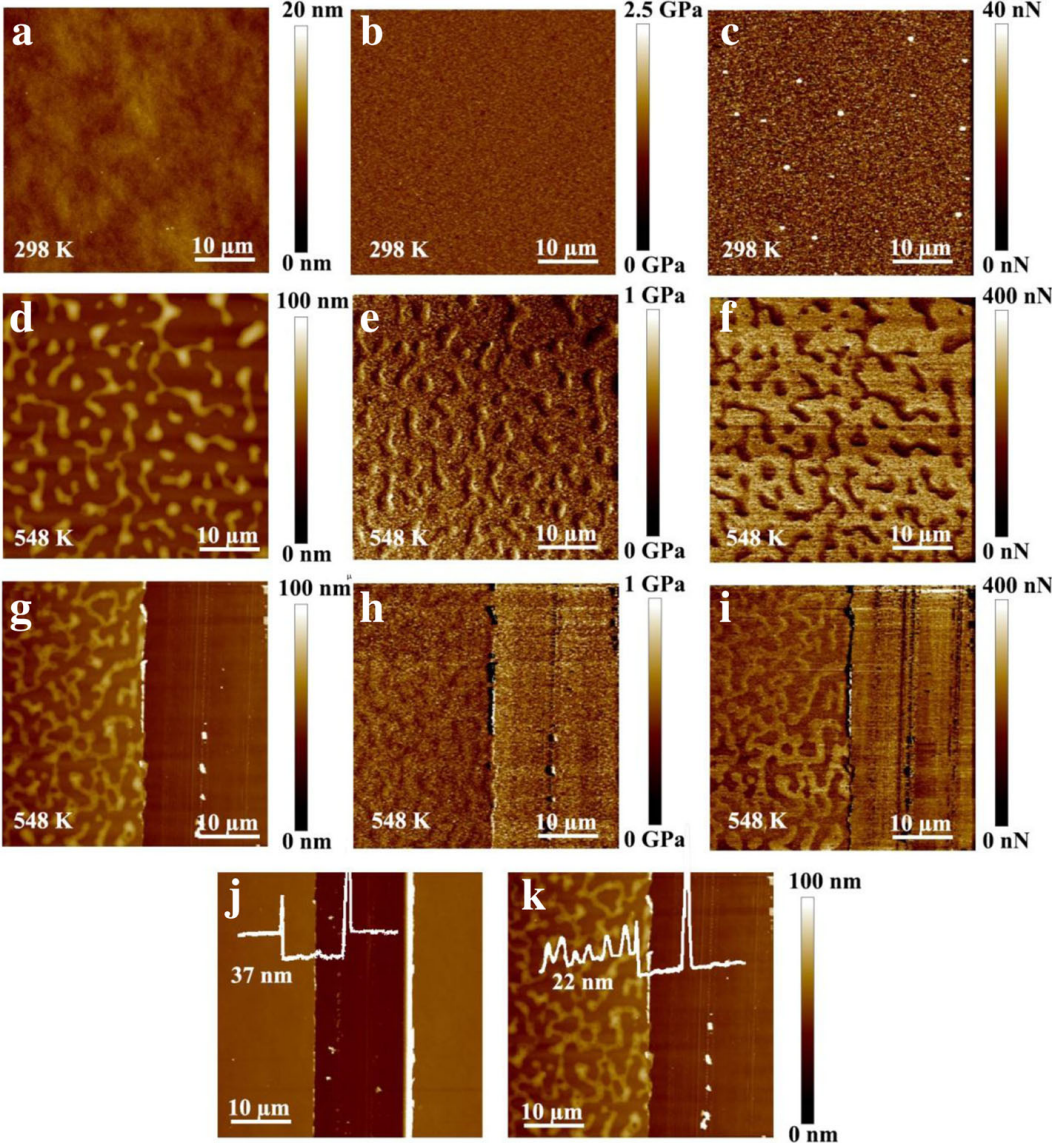
The surface morphology **a**, modulus mapping **b**, and adhesion force mapping **c** of PS-PMMA blends at 298 K; the surface morphology **d**, **g**, modulus mapping **e**, **h**, and adhesion force mapping **f**, **i** of PS-PMMA blends at 548 K; AFM topography of the thickness for PS-PMMA blends under different temperatures: 298 K **j** and 548 K **k**

In our previous work, the relaxation dynamics and glass transition temperature of ultrathin PS or PMMA films were in situ studied by monitoring the surface potential. We found that the *T*_*g*_ of ultrathin polymer films is clearly independent of film thickness, and the *T*_*g*_ of ultrathin PS and PMMA films were 328 and 358 K, respectively. In order to intuitively observe the difference between PS and PMMA films, PS-PMMA blend solution was spin-coated on Si substrate to form polymer films. The morphology, modulus and adhesion mapping were measured under different temperatures in Fig. 2. At 298 K, the property differences of PS/PMMA were not obvious in Fig. 2a–c. However, when the temperature increased to 548 K, the chain segment relaxation behavior was occurred for normal thin PS films, and then, the dewetting phenomenon was obtained compared to normal thin PMMA films. The initial film thickness of PS-PMMA blends was 37 nm in Fig. 2j. When normal thin PS chains were easily dewetted and removed from normal thin PMMA films, the film thickness reduced to 22 nm in Fig. 2k. The contrast of modulus and adhesion force between PS-PMMA blends was significant in Fig. 2h, i. The change of modulus and adhesion force mapping versus temperature was qualitatively estimated. In order to quantitatively calculate the adhesion force under different temperatures, we collected the force curves of normal thin PS films. According to the discontinuous change of the adhesion force with temperature, the *T*_*g*_ of normal thin PS film was calculated.

## Results and Discussion

As mentioned above, FFM could be employed to detect the molecular motion in thin polymer films, because the friction properties of polymer films are closely related to viscoelasticity in the horizontal orientation [17]. Compared to the friction force, adhesion force emphasizes on the reflection of the mechanical properties of thin polymer films in the vertical direction [23]. Moreover, the adhesion force is acquired from the interested point (place) by monitoring the cantilever reflection, while the measurement of friction force requires scanning the whole sample. Hence, the interference from the substrate is relatively small, and there is only interaction between tip and sample either for hard or soft samples [21]. The *F*_ad_ is acquired by recording the force-distance curves, and the mechanical properties of normal thin polymer surface are deduced from the changes in the slope of force-distance curve.

**Fig. 3:**
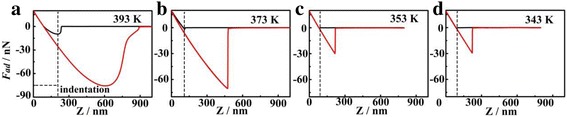
Force-distance curves of normal thin PS films with a thickness of 93 nm obtained at different temperatures: **a** 393 K, **b** 373 K, **c** 353 K, and **d** 343 K. The distance from dashed line to 0 nm (horizontal coordinate) represents the indentation depth

Temperature dependences are considered to be crucial to polymer relaxation behavior, especially at segmental level, because the polymer main chains in thin films will evolve from non-equilibrium toward equilibrium [13]. Hence, the changes in polymer caused by temperature stimulation could induce the variation of viscoelasticity of polymer films. In order to directly illustrate the influence of temperature on the adhesion force, force-distance curves at different temperatures are recorded. An in situ heater/cooler device is employed to obtain well-controlled temperature. The measurement of *T*_*g*_ was commonly conducted during cooling down process because the glass transition process transited from non-equilibrium to equilibrium. It is reported in the literature that there is no difference for the measurement at the same temperature but during the different processes, e.g., heating up and cooling down. The temperature changing direction is cooling down from a temperature higher than bulk *T*_*g*_. The temperature interval is 10 K, and the cooling down rate is 2 K/min. Each temperature is kept for 5 min to obtain thermal equilibration. The pull-off force, which is regarded as the adhesion force (*F*_ad_), is measured at the temperatures of 393, 373, 353, and 343 K for thin PS films with a thickness of 93 nm, as shown in Fig. 3. At a relatively higher temperature of 393 K, the force curve shows a distinctive tail, which is corresponding to a softer surface. A larger indentation of 208 nm is observed, which is illustrated by the dashed line. With the temperature decreasing, the force curve is approaching a standard force curve, and the indentation decreases to 109 nm for 373 K and 89 nm for 353 K. When the temperature decreases to 343 K, a very standard force curve for a stiff surface is captured with an indentation of 89 nm, which indicates that the interaction between tip and sample is weaker.

**Fig. 4:**
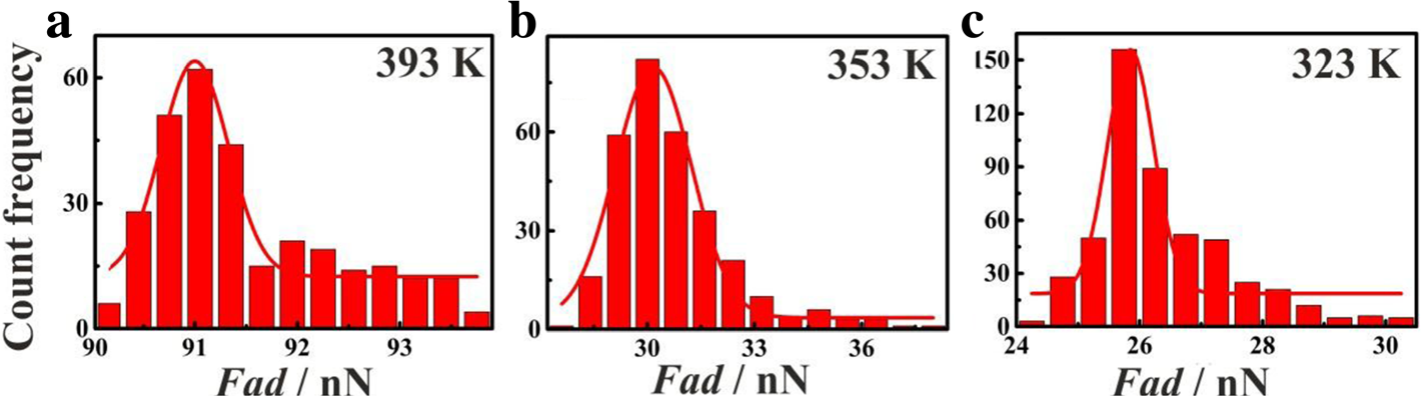
The histogram of adhesion force between AFM tip and sample under different temperatures: **a** 393 K, **b** 343 K, and **c** 303 K

A number of force curves (300) are captured, and the adhesion forces are calculated accordingly. Statistics and frequency counts are conducted to eliminate the random factors. Reliable tip-sample interaction force spectrum is obtained for thin PS film with a thickness of 93 nm under different temperatures, as shown in Fig. 4. The *F*_ad_ measured at 393, 353, and 323 K are 91, 30, and 26 nN, respectively.

**Fig. 5:**
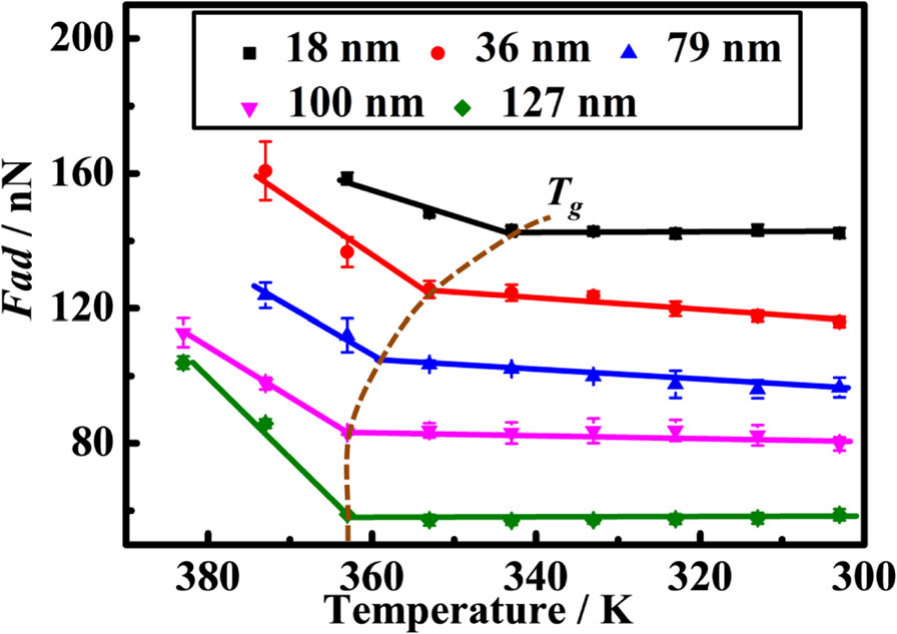
Temperature dependence of the adhesion force for normal thin PS films with different thicknesses from 18 to 127 nm

The temperature dependences of *F*_ad_ for normal thin PS films with different thicknesses are shown in Fig. 5. The film thicknesses of normal thin PS films are controlled between 18 and 127 nm, which are regarded as normal thin polymer films. Linear decrease curves are obtained for normal thin PS films in the initial stage. At a temperature higher than the *T*_*g*_ of normal thin PS films, the structural relaxation caused by cooperatively rearranging regions of tens to hundreds of repeating units is more pronounced. The relaxation dynamics is always associated with the α-relaxation with large scale motions of segmental mobility [13]. The elasticity of the film surface is more pronounced during this period, and the mechanical properties show obvious elasticity, resulting in larger adhesion force.

When the temperature decreases, the thermal motion of polymer main chains is slowed down, and an abrupt transition point could be obtained. With temperature further decreasing, the adhesion forces tend to be steady for normal thin PS films with different thicknesses. During this period, the elastic normal thin films start to transfer to glassy status, and a lower adhesion force is observed, which may be associated with a variety of smaller scale dynamics [13, 24]. It should be noted that the small-scale sub-segmental relaxations including the orientation of ester side groups are difficult to be characterized using other traditional techniques. The point at which the two straight lines intersect is the discontinuity in adhesion force measurement, and the discontinuity point is regarded as the *T*_*g*_ of normal thin polymer film, which is reported in previous study [5, 25–27].

**Fig. 6:**
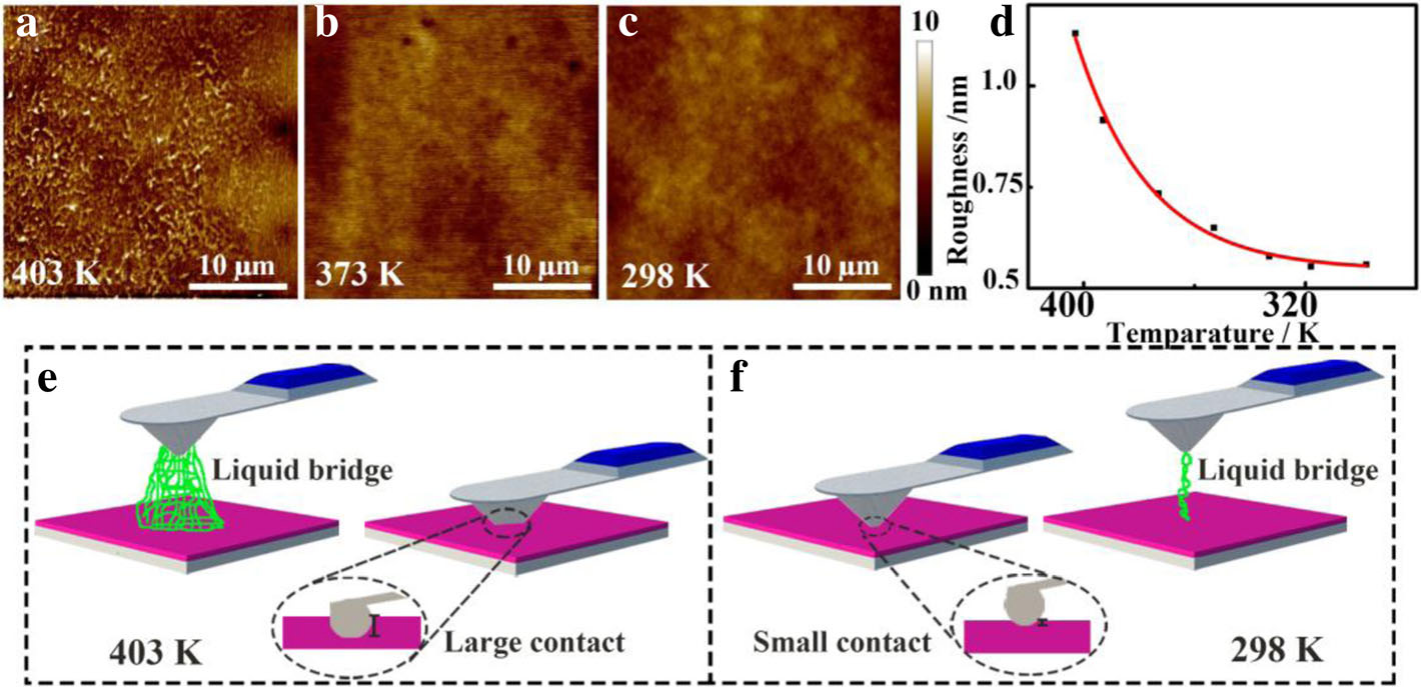
AFM topography images of normal thin PS films at different temperatures: **a** 403 K, **b** 373 K, and **c** 298 K. **d** Temperature dependence of roughness obtained for normal thin PS film with a thickness of 20 nm. Diagram of adhesion force variation for normal thin PS films under different temparatures: **e** 403 K and **f** 298 K

Commonly, the adhesion force between AFM tip and film surface is contributed by several forces, including contact forces, van der Waals forces, capillary force, and electrostatic forces. Where the van der Waals force is constant in this situation, there is no electrostatic force because no external voltage is applied. Thus, the main contribution to the adhesion force is the contact force and the capillary force [28]. As mentioned above, the depth of tip indentation into sample surface reflects the viscoelasticity status of polymer films and the contact area, which could be characterized by scanning surface morphology variations [28]. The topographies of thin PS films of 20 nm are captured by AFM during a cooling down process, as shown in Fig. 6a–c. The roughness of normal thin PS films under different temperatures decreases from 1.13 to 0.56 nm, as shown in Fig. 6d. It could be observed that the morphology of thin PS films is rougher at high temperature of 403 K, which is higher than the bulk *T*_*g*_. At this stage, the rougher surface and the softer surface of normal thin PS films could induce a larger tip indentation, which causes increasing the real contact area between the AFM tip and surfaces. As has been reported, the adhesion force is proportional to the real contact area between surface asperities [22, 29]. Therefore, the greater contact area results in the greater contact force. Moreover, the active polymer main chains movement also attributes to the forming of a viscous liquid as a liquid bridge [15], causing a large liquid bridge force. Ultimately, the larger contact force and the liquid bridge force contribute to a large adhesion force at high temperature.

With temperature decreasing, the movement of polymer chains is slowed down, and the morphology is approaching a glassy status. Due to a flat surface and low indentation depth, the contact area between AFM tip and sample surface is relatively small and invariable, and the frozen polymer chains will induce a low capillary force when the relative humidity is controlled very low. Hence, the adhesion force between AFM tip and normal thin PS film surface is relatively low and keeps constant. The schematic illustration of the contribution of contact area and liquid bridge to the *F*_ad_ is shown in Fig. 6e, f.

**Fig. 7:**
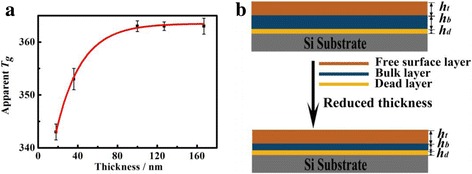
**a** The film thickness dependence of *T*_*g*_ for normal thin PS films during the cooling down process. **b** Schematic illustration of the reduction of *T*_*g*_ with film thickness for normal thin polymer films in three-layer model

The *T*_*g*_ of normal thin PS films with different film thicknesses are calculated and illustrated in Table 1. The *T*_*g*_ of bulk PS measured by differential scanning calorimetry is 363 K. According to Table 1, the *T*_*g*_ is kept constant (equal to the value of bulk *T*_*g*_) for thicker PS films (larger than 100 nm), which is in agreement with previous reports [13]. However, the apparent *T*_*g*_ of normal thin PS films shows obvious thickness dependence when the film thickness is lower than 100 nm, which is also regarded as normal thin films. The apparent *T*_*g*_ of normal thin PS film decreases with film thickness reducing as shown in Fig. 7a.

**Table 1:** The *T*_*g*_ of Normal Thin PS Films with Various Thicknesses

Film Thickness/nm	18	79	93	100	127	167
Transition Temperature/K	343	353	358	363	363	363

An empirical equation of the film thickness dependence of *T*_*g*_ was proposed by Keddie et al. as the following [5]:

1 $$ {T}_g(d)={T}_g\left(\mathrm{bulk}\right)\left[1\hbox{-} {\left(\frac{A}{d}\right)}^{\updelta}\right] $$

where *T*_*g*_(*d*) is the measured glass transition temperature of the polymer film; *T*_*g*_(bulk) is the *T*_*g*_ of the bulk material; *A* is the characteristic length equal to 3.2 ± 0.6 nm, and the exponent *δ* = 1.8 ± 0.2.

From the empirical equation, it could be obtained that the *T*_*g*_(*d*) is approaching the *T*_*g*_(bulk) when the film thickness was much larger than the characteristic length. Two-layer and three-layer models [4, 5, 30–32] have been proposed to explain the *T*_*g*_ depression phenomenon of nanoconfined thin polymer films [1, 27, 32]. The top layer both in two models is regarded as a liquid-like layer, which could enhance the mobility of the polymer chain and hence reduce the *T*_*g*_ of polymer films.

In order to illustrate the dependence of the film thickness and *T*_*g*_, the three-layer model is introduced in this study, in which substrate supported thin polymer film contains three layers. As shown in Fig. 7, the thickness of the top layer, middle layer and bottom layer is defined as *h*_*t*_, *h*_*b*_*,* and *h*_*d*_ respectively. The interface layer between the polymer and the substrate is a dead layer, which shows no mobility due to interaction force between sample and substrate [4]. The middle layer is bulk-like layer, which has similar behaviors with the bulk materials. Besides, the top layer of the film is the free surface layer, which enhances the mobility of polymer main chains [31, 33]. For thick polymer films, the relaxation of polymer main chains occurs at a higher temperature, where the interfacial effect is of domination, and the movement of molecular chain on the substrate is highly depressed, resulting in the constant *T*_*g*_ [17]. The existence of liquid-like layer in normal thin PS film leads to the reduction of the apparent *T*_*g*_ [34, 35], in which the mobility of the polymer surface is larger than that of the bulk matrix [17, 36], and the relaxation of polymer chain segment at relatively low temperature. Polymer chain ends at the air-polymer interface tends to move toward the surface, which leads to the increase of free volume and acceleration of chain mobility. The *h*_*b*_ reduced with the film thickness further decreasing, in which the conformation transition of free surface layer extends into the bulk matrix, resulting in the enhancement of the total region molecular chains mobility [36]. Therefore, when film thickness decreases, the relative fraction of *h*_*t*_ to total *h* increases and leads to an overall decrease of *T*_*g*_ in normal thin PS films. Thus, the *T*_*g*_ of normal thin PS films is reduced with decreasing film thickness.

## Conclusions

To sum up, in this study, the elasticity properties of thin polymer films are characterized by in situ capturing the variation of force curves, which is more sensitive due to the high resolution of AFM tip. The adhesion force, *F*_ad_, which originates from the very trifle variation of the interaction between the AFM tip and the surface, could quantitatively reflect the mechanical properties of the normal thin polymer films. The *T*_*g*_ of normal thin PS film was successfully calculated by the abrupt variation of *F*_ad_ under temperature stimulation. Moreover, the film thickness dependence of *T*_*g*_ for normal thin PS films is calculated by monitoring the adhesion force variations. The study illustrates that the *T*_*g*_ of normal thin PS films supported on silicon decreases with the film thickness reducing. This phenomenon is consistent with our previous work [37], in which the *T*_*g*_ of normal thin PS films depresses with decreasing film thickness. A consistent interpretation of the result is possible by the existence of liquid-like layer enhancing the mobility of polymer main chains. The result could be beneficial for understanding the relaxation dynamics of normal thin polymer films. However, more studies are needed to conduct for quantitative measurement due to many controversies about thickness dependence of *T*_*g*_ for normal thin polymer films.

## Abbreviations

AFM: Atomic force microscopy
AFMAM: Atomic force microscopic adhesion measurement
EFM: Electric force microscopy
*F*_*ad*_: Adhesion force
FFM: Friction force microscopy
LFM: Lateral force microscopy
PS: Polystyrene
PtBuA: Poly (tert-butyl acrylate)
SFM: Scanning force microscopy
*T*_*g*_: Glass transition temperature

## References

[1] Yang Z, Clough A, Lam CH, Tsui OKC (2011). Glass transition dynamics and surface mobility of entangled polystyrene films at equilibrium. Macromolecules.

[2] Hammerschmidt JA, Gladfelter WL, Haugstad G (1999). Probing polymer viscoelastic relaxations with temperature-controlled friction force microscopy. Macromolecules.

[3] Fakhraai Z, Forrest JA (2008). Measuring the surface dynamics of glassy polymers. Science.

[4] DeMaggio GB, Frieze WE, Gidley DW, Zhu M, Hristov HA, Yee AF (1997). Interface and surface effects on the glass transition in thin polystyrene films. Phys Rev Lett.

[5] Keddie JL, Jones RAL, Cory RA (1994). Size-dependent depression of the glass transition temperature in polymer films. Europhys Lett.

[6] Tsui OKC, Russell TP, Hawker CJ (2001). Effect of interfacial interactions on the glass transition of polymer thin films. Macromolecules.

[7] Riedel C, Sweeney R, Israeloff NE, Arinero R, Schwartz GA, Alegria A, Tordjeman P, Colmenero J (2010). Imaging dielectric relaxation in nanostructured polymers by frequency modulation electrostatic force microscopy. Appl Phys Lett.

[8] Nguyen HK, Prevosto D, Labardi M, Capaccioli S, Lucchesi M, Rolla P (2011). Effect of confinement on structural relaxation in ultrathin polymer films investigated by local dielectric spectroscopy. Macromolecules.

[9] Revilla RI, Guan L, Zhu XY, Quan BG, Yang YL, Wang C (2012). Electrowetting phenomenon on nanostructured surfaces studied by using atomic force microscopy. J Phys Chem C.

[10] Masubuchi S, Arai M, Machida T (2011). Atomic force microscopy based tunable local anodic oxidation of graphene. Nano Lett.

[11] Koivistoinen J, Sládková L, Aumanen J, Koskinen P, Roberts K, Johansson A, Myllyperkiö P, Pettersson M (2016). From seeds to islands: growth of oxidized graphene by two-photon oxidation. J Phys Chem C.

[12] Zhao D, Peng JX, Tang XF, Zhang DD, Qiu XH, Yang YL, Wang YP, Zhang MN, Guan L, Cao TB (2013). Charge-induced local dewetting on polymer electrets studied by atomic force microscopy. Soft Matter.

[13] Lin ZH, Gao D, Guan L, Zhang M, Zhu XY, Yang YL, Qiu XH, Zhang JP (2016). Charge-pattern indicated relaxation dynamics and glass transition of polymer thin films studied by atomic force microscopy. J Phys Chem C.

[14] Yang Z, Fujii Y, Lee FK, Lam CH, Tsui OKC (2010). Glass transition dynamics and surface layer mobility in unentangled polystyrene films. Science.

[15] Marti O, Stifter T, Waschipky H, Quintus M, Hild S (1999). Scanning probe microscopy of heterogeneous polymers. Colloid Surf A.

[16] Hammerschmidt JA, Moasser B, Gladfelter WL, Haugstad G, Jones RR (1996). Polymer viscoelastic properties measured by friction force microscopy. Macromolecules.

[17] Akabori K, Tanaka K, Kajiyama T, Takahara A (2003). Anomalous surface relaxation process in polystyrene ultrathin films. Macromolecules.

[18] Cappella B, Kaliappan SK, Sturm H (2005). Using AFM force−distance curves to study the glass-to-rubber transition of amorphous polymers and their elastic−plastic properties as a function of temperature. Macromolecules.

[19] Bliznyuk VN, Assender HE and Briggs GAD (2002) Surface glass transition temperature of amorphous polymers. A new insight with sfm Macromolecules 35: 6613-6622

[20] Wang XP, Xiao XD, Tsui OKC (2001). Surface viscoelasticity studies of ultrathin polymer films using atomic force microscopic adhesion measurements. Macromolecules.

[21] Sun YJ, Akhremitchev B, Walker GC (2004). Using the adhesive interaction between atomic force microscopy tips and polymer surfaces to measure the elastic modulus of compliant samples. Langmuir.

[22] Revilla R, Guan L, Zhu XY, Yang YL, Wang C (2011). Nanoscale electrowetting effects observed by using friction force microscopy. Langmuir.

[23] Tsui OKC, Wang XP, Ho JYL, Ng TK, Xiao XD (2000). Studying surface glass-to-rubber transition using atomic force microscopic adhesion measurements. Macromolecules.

[24] Struik LCE (1977). Physical aging in plastics and other glassy materials. Polym Eng Sci.

[25] Ellison CJ, Torkelson JM (2003). The distribution of glass-transition temperatures in nanoscopically confined glass formers. Nat Mater.

[26] Keddie JL, Jones RAL, Cory RA (1994). Interface and surface effects on the glass-transition temperature in thin polymer films. Faraday Discuss.

[27] Singh L, Ludovice PJ, Henderson CL (2004). Influence of molecular weight and film thickness on the glass transition temperature and coefficient of thermal expansion of supported ultrathin polymer films. Thin Solid Films.

[28] Jones R, Pollock HM, Cleaver JAS, Hodges CS (2002). Adhesion forces between glass and silicon surfaces in air studied by AFM: effects of relative humidity, particle size, roughness, and surface treatment. Langmuir.

[29] Butt HJ, Cappella B, Kappl M (2005). Force measurements with the atomic force microscope: technique, interpretation and applications. Surf Sci Rep.

[30] Forrest JA, Dalnoki-Veress K (2001). The glass transition in thin polymer films. Adv Colloid Interface.

[31] Fukao K, Miyamoto Y (2000). Glass transitions and dynamics in thin polymer films: dielectric relaxation of thin films of polystyrene. Phys Rev E.

[32] Miyazaki T, Nishida K, Kanaya T (2004). Thermal expansion behavior of ultrathin polymer films supported on silicon substrate. Phys Rev E.

[33] Paeng K, Richert R, Ediger MD (2012). Molecular mobility in supported thin films of polystyrene, poly(methyl methacrylate), and poly(2-vinyl pyridine) probed by dye reorientation. Soft Matter.

[34] Forrest JA, Dalnoki-Veress K, Stevens JR, Dutcher JR (1996). Effect of free surfaces on the glass transition temperature of thin polymer films. Phys Rev Lett.

[35] Forrest JA, Mattsson J (2000). Reductions of the glass transition temperature in thin polymer films: probing the length scale of cooperative dynamics. Phys Rev E.

[36] Vignaud G, Chebil MS, Bal JK, Delorme N, Beuvier T, Grohens Y, Gibaud A (2014). Densification and depression in glass transition temperature in polystyrene thin films. Langmuir.

[37] Qian XQ, Lin ZH, Guan L, Li Q, Wang YP, Zhang MN, Dong MD (2017). In situ probing the relaxation properties of ultrathin polystyrene films by using electric force microscopy. Nanoscale Res Lett.

